# Surgical Release of Severe Flexion Contracture for Oncologic Knee Arthroplasty

**DOI:** 10.2174/1874325001711010045

**Published:** 2017-02-24

**Authors:** Vincent Y. Ng, Philip Louie, Stephanie Punt, Ernest U. Conrad

**Affiliations:** 1 University of Maryland Medical Center - Orthopaedics 110 S. Paca St, 6^th^ Floor, Baltimore, Maryland 21201, United States; 2 Rush University - Orthopaedics, Chicago, United States; 3 University of Washington - Orthopedics and Sports Medicine 1959 NE Pacific Street , Seattle, Washington 98195, United States

**Keywords:** Flexion contracture, Megaprosthesis, Release, Oncologic, Sarcoma, Revision

## Abstract

**Background:**

Severe postoperative knee contractures after arthroplasty or megaprosthesis reconstruction occur rarely, but are devastating complications. Management of preoperative flexion contractures is well-described, but there is a paucity of literature for surgical treatment of postoperative contractures.

A retrospective chart review was performed for a single surgeon of cases between 1996 and 2014.

**Results:**

Nine patients (5 of 66 for pediatrics; 4 of 95 for adults) underwent surgical release for severe stiffness after implantation of knee megaprosthesis. The total arc of motion was improved from a preoperative mean of 34° (range, 10° to 70°) to a postoperative mean 89° (63° to 125°). The amount of extension improved by a mean of 27° (range, -3° to +70°) and the amount of flexion improved by a mean of 28° (range, -10° to +75°).

**Conclusion:**

Surgical release of severe postoperative knee contracture is a challenging procedure, but in most cases, the amount of extension and flexion can be improved, yielding a greater total arc of motion.

## INTRODUCTION

Severe postoperative flexion contractures after oncologic megaprosthesis reconstruction occur in 1-10% of cases, but are a serious complication because of the associated functional results [1, 2]. Studies have demonstrated mild flexion contractures of about 10° after primary total knee arthroplasty tend to improve even several years postoperatively and have limited long-term functional compromise [3, 4]. The energy cost of walking, however, is significantly increased with contractures greater than 20°^5^. There are established treatment protocols and algorithms for addressing preoperative flexion contractures [5, 6], but there is a paucity of literature for surgically addressing severe contractures postoperatively, particularly those involving megaprostheses. Nonsurgical measures such as physical therapy, manipulation under anesthesia and arthroscopic or minimal releases are only effective in mild cases [7]. This article on one surgeon’s technique, on 11 patients over 18 years, describer an effective method for the surgical release of severe contractures following oncologic arthroplasty of the knee. To date, similar surgical technique guidelines for arthroplasty-related flexion contractures of the knee have not been published.

## PREOPERATIVE PLANNING

Indications for surgical consideration include at least 6 months of formal physical therapy and dynamic splinting with no significant improvement and flexion contractures greater than 25° that impede patient activity. The patient and family are thoroughly counseled on the risks of surgery particularly the risk of permanent injury to major nerves and vessels, specifically the popliteal artery and vein, and the tibial and common peroneal nerves. The risk of implant infection, wound complications, or recurrent contracture of the knee are also discussed thoroughly and documented preoperatively. Inflammatory markers including erythrocyte sedimentation rate and C-reactive protein are assessed preoperatively to rule out an indolent infection. The importance of postoperative physical therapy is emphasized for the success of the procedure. A very careful neurovascular experiment is performed and documented for comparison in the case of any postoperative deficits. The pre-- and post--operative range of motion (ROM) are carefully recorded with a goniometer.

## SURGICAL TECHNIQUE

Most patients receive either an epidural or a sciatic and femoral pain catheter to assist analgesia. Effectively controlling postoperative pain is important for patients to be able to tolerate early postoperative range of motion and physical therapy. No neuromuscular blocking agents are used in order to allow the detection of encroachment on nerves while using electrocautery. No tourniquet is used.

A long medial or lateral parapatellar approach is followed in line with the patient’s prior surgical incision. It is important that the same previous exposure through the knee joint and quadriceps is utilized to minimize damage to the extensor mechanism. The femoral nerve arborizes within the proximal third of the thigh and should be carefully preserved if the dissection is extended to the proximal thigh. Due to prior resection, critical anatomic structures may be scarred or located in atypical positions and surgical dissection should be performed with care.

The anteromedial arthrotomy of the knee extends distally to the proximal tibia and the pes anserinus and medial soft tissues are elevated off the tibial metaphysis. The tibia can be externally rotated and the patella everted. The distal femoral oncologic component is disengaged from the proximal tibial component and the areas of reactive scar tissue are excised. The distal femoral component condyles and body components should be disarticulated to facilitate exposure to the popliteal fossa and posterior aspects of the knee and proximal tibia. Soft tissue adhesions to the distal posterior femur at the implant “collar” are released with a release of the posterior margin of vastus lateralis and vastus medialis. In a standard arthroplasty setting, the origins of the gastrocnemius muscles are elevated. There may be several superior geniculate vessels that need to be ligated at this step.

With the patella everted, a lateral patellar release is performed, starting from the level of the inferior pole of the patella and traveling proximally to 15 cm. Electrocautery is used to incise the complete thickness of the pseudocapsule formed within the joint until the undersurface of vastus lateralis is observed, avoiding incision of the skin superficially.

Release of the posterior margin of the vastus medialis in the popliteal fossa is challenging due to the proximity of the femoral artery at the “adductor hiatus”, approximately 12-14 cm from the knee joint. A sterile doppler probe is used to help locate the popliteal artery before deep fascial release in that location. In this revision setting, there is usually a thick posterior pseudocapsule typically 1-2 cm thick. The popliteal artery is the most anterior of the neurovascular structures in the posterior knee. At the level of the knee joint, it is located in the posterior midline coursing into the posterior calf. More proximally, approximately 8-10 cm above the patella in an adult, the femoral artery dives posterior to the posteromedial intermuscular septum and through the adductor hiatus. It lies directly posterior to a thin layer of adductor hiatus tissue and typically courses posterior to the femur, immediately posterior to the fascial attachment of the compartment posterior to the medial femoral diaphysis. The most distal perforating branch of the deep femoral artery perforates the lateral intermuscular septum at this level as well. If the patient had a prior distal femoral replacement, the distal adductor magnus insertion has already been released from the femur. The doppler is used to find the vessel which may be in an atypical location due to scarring. Placing the probe through the posteromedial release allows one to auscultate the vessel behind the thick posterior pseudocapsule. Locating the popliteal artery with a doppler before deep dissection in this location is important in order to minimize the risk of incidental laceration of popliteal vessels.

Once the approximate path and depth of the popliteal artery at the posterior margin of the vastus medialis and within the popliteal fossa are determined with the doppler, the posterior capsule and posteromedial vastus medialis can be resected or released. Because the depth of the artery is known, the posterior capsule can also be thinned by using electrocautery to remove the more anterior or superficial layers of scar. The posterior capsule should be released longitudinally at its posteromedial and posterolateral margins to reduce tension on the closure as the medial and lateral tissues are brought anteriorly Figs. (**1**, **2**).

The posterolateral dissection of the popliteal fossa has the common peroneal nerve at its distal margin approximately 6-8 cm proximal to the fibular head and usually posterior to the biceps femoris tendon. Dissection through the area of fat directly posterior to the biceps femoris usually reveals the common peroneal nerve which is located posterior and lateral to the popliteal vessels. Protecting the peroneal nerve, the biceps femoris can be fractionally lengthened with partial tenotomies or step--cuts at several levels. The tibial nerve should be identified adjacent and lateral to the popliteal artery in the popliteal fossa.

The proximal tibia should be dissected at the posterior capsular attachment for 1-2 cm, staying proximal to the soleal arch to confirm the location of the popliteal artery with the doppler at the level of the tibial joint line. Following this, the soft tissues are elevated off the posterior proximal tibia medial to the artery. This includes the major insertion of semimembranosus and the origin of popliteus.

If available, the use of a smaller condylar body component is helpful in revision for relaxing the extensor mechanism and increasing flexion. It also facilitates tissue closure if the patient is at risk for wound-healing complications. Shortening of the intercalary segments may be considered, but is typically not sufficient for significant correction of a severe flexion contracture.

Full extension should be achieved at this point. Doppler is used to auscultate the dorsalis pedis and anterior tibial pulse at the ankle to confirm full extension without undue tension on the popliteal vessels. Moreover, the maximal extension and the adequacy of the distal pulses are analyzed to ensure that there is no vascular compromise with complete extension. The location of the distal pulses is marked to facilitate postoperative assessments.

Deep drains are placed to prevent hematoma and promote incisional wound healing. Any adhesive dressings are placed with the knee in flexion, not in extension, to prevent blistering of the skin with ROM. A well-padded dressing is applied with an anterior and posterior plaster splint with the knee in full extension as long as no compromise of the distal pulses is noted during the surgery using the doppler with the knee in full extension. Postoperative Protocol:

Patients are kept on prophylactic intravenous antibiotics until the intraoperative cultures return negative by the postoperative day 5 and the drains have minimal output and are removed by postoperative day 2 to 5. Aspirin 325 mg twice daily, early mobilization, and sequential compression devices are used to prevent venous thrombus. If there is any concern for vascular injury, an ultrasound study can be performed in the early postoperative period.

The postoperative splint is kept in place for two days except if the patient experiences paresthesias, ischemic pain, or poor perfusion; in this case, the splint is removed and the knee is flexed. On the third day, the splint is removed and a continuous passive motion (CPM) machine is started at a 70-80° arc of motion and increased by 5-10° each day. If the patient has a severe (>40°) preoperative flexion contracture, the postoperative splint may be left in place for 5-7 days before beginning the ROM. The CPM machine should be used for at least two hours every 4-6 hours for the first week postoperatively. A spring-loaded dynamic splint or extension splint may be used to maintain extension at night after discharge for 4-6 weeks. Physical therapy is started on the postoperative day 1 for mobilization and gait training, and continued along with the CPM and dynamic splint for the ROM.

## RESULTS

Based on a retrospective chart review, 11 patients with a megaprosthesis about the knee and limited postoperative range of motion despite aggressive conservative management underwent surgical release for severe contracture between 1996 and 2014 by a single surgeon. Two patients were excluded from the review due to inadequate documentation or tumor recurrence leading to amputation or death. Nine patients (5 pediatric; 4 adult) with 9 tumors were included with a mean follow-up of 22 months (range, 2 to 69 mos). The incidence of flexion contracture requiring surgery was 8% and 4% in pediatric (<18 years old) and adult patients, respectively. The total arc of motion was improved from a preoperative mean of 34° (range, 10° to 70°) to a postoperative mean 89° (63° to 125°), an average difference of 55°. The amount of extension improved by a mean of 27° (range, 3° worsening to 70° improvement) and the amount of maximum flexion improved by a mean of 28° (range, 10° worsening to 75° improvement). No vascular or nerve--related complications occurred and one patient developed cellulitis treated with antibiotics.

## CONCLUSION

Surgical release of severe postoperative knee contracture is a challenging procedure, but in most cases, the amount of extension and flexion can be improved, yielding a greater total arc of motion. It is crucial to discuss the risks of revision surgery in patients with megaprostheses particularly infection, injury to nerves or vessels, and recurrent contractures.

Limitations of this chart review include a relatively short follow-up and small number of patients. Despite numerous studies on mild flexion contractures after primary knee arthroplasty [3, 4], there are no other described techniques for the release of severe flexion contractures for megaprostheses.

This technique is a useful tool in the armamentarium of orthopaedic surgeons with patients requiring complex arthroplasty revisions or megaprosthetic reconstruction.

The rotating tibial platform has been disengaged from all-polyethylene tibial components to allow delivery of the distal femur out of the wound. The posterior capsule is thinned in the coronal plane and longitudinal releases are performed (white arrows) after the path of the popliteal artery is ascertained by doppler to locate the peroneal nerve.

Coronal and sagittal diagrams show the location of posterior tissue releases along with the posterior edge of vastus medialis and lateralis. The femoral artery and common peroneal nerves are denoted by the red and brown lines, respectively. The hashed lines denote the location of the posterior capsule division and the arrows show where the posterior capsule is elevated off the posterior aspect of the femur and tibia.

## Figures and Tables

**Fig. (1):**
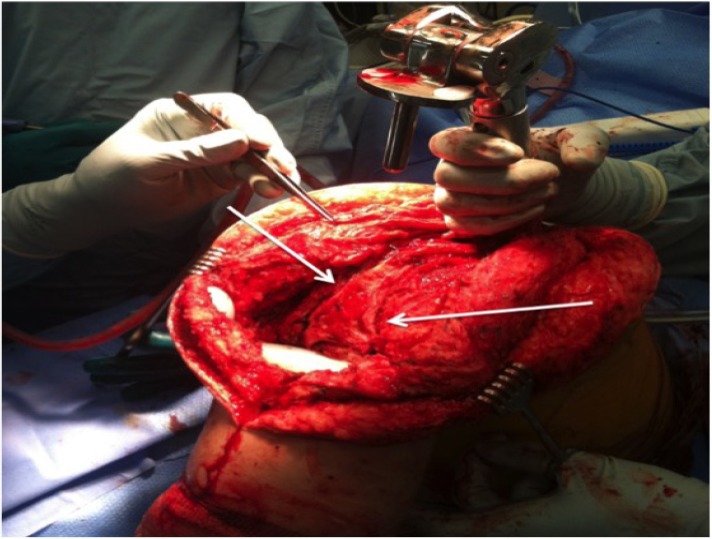
Release of Posterior Capsule.

**Fig. (2):**
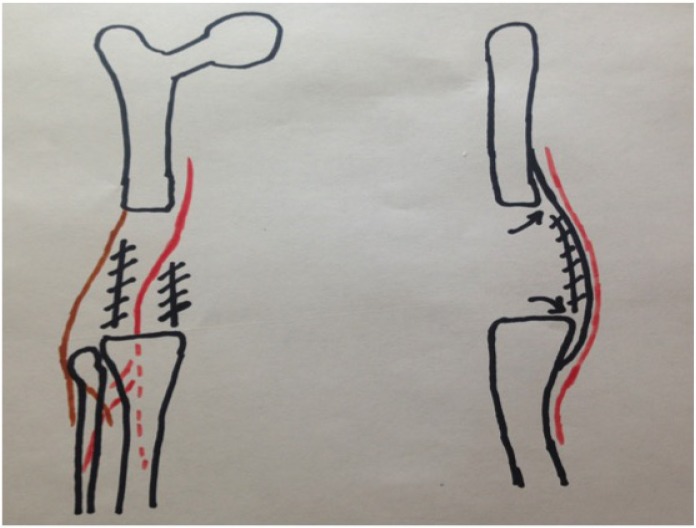
Posterior tissue releases.

## LIST OF ABBREVIATIONS

CPM: = Continuous passive motion
ROM: = Range of motion
